# Supplementary material for: Patterns of Intron Gain and Loss in Fungi
Source: PLoS Biol. 2004 Nov 30;2(12):e422. doi: 10.1371/journal.pbio.0020422 (PMC532390; doi:10.1371/journal.pbio.0020422)
Supplement: Table S1 — Also available at http://genes.mit.edu/NielsenEtAl/. (4.3 MB ZIP). [file pbio.0020422.st001.zip › NielsenEtAl/html/380.html]

AN9160.1.NCU03023.1.MG01605.1.FG00489.1


```
 CLUSTAL W (1.82) Multiple Sequence Alignments - Introns Inserted


Sequence 1: NCU03023.1	656 aa
Sequence 2: MG01605.1	652 aa
Sequence 3: FG00489.1	658 aa
Sequence 4: AN9160.1	897 aa
Alignment Length: 943 aa
Number Identitical Residues: 253 aa
Alignment Score (without introns) 15061


MG01605.1 	---~--------------------------------------------------------
NCU03023.1	---~--------------------------------------------------------
FG00489.1 	---~--------------------------------------------------------
AN9160.1  	MSK0FSLKGRTALVTGGGRGCGLAFARGLAEAGADVAVFDIIDPRPEFHAIEKDYNVRTA
          	 :.  : .. ::  :... ... : : . :.:.:. :  .  .. ..  : ... .  ::

MG01605.1 	---~--------------------------------------------------------
NCU03023.1	---~--------------------------------------------------------
FG00489.1 	---~--------------------------------------------------------
AN9160.1  	YYK2VDVSSQDSLKDGFAQFQQDFDGKLDICVPCAGINRHTCFLGFTYEEHHDLLSINVL
          	  .  . ::..: ... :. ... ... . . ..:. .  :.  . : ..  .  : .  

MG01605.1 	------------------------------------------------------------
NCU03023.1	------------------------------------------------------------
FG00489.1 	------------------------------------------------------------
AN9160.1  	GLYFTAQYAAKQMIANGTHHGSIILVASMASYVAVRDQNCSAYCGTKGAVRAMCPAIAKE
          	.   ::. ::..  :..:  .:    :: ::  :  ....:: ..:..:  : ..: :..

MG01605.1 	--------------------------------------------------~---MAPSVL
NCU03023.1	--------------------------------------------------~---MAPGVL
FG00489.1 	--------------------------------------------------~---MVPSVV
AN9160.1  	LTEYGIRVNSISPGYVNTEMTAAFSHLVEGWKAETINKRIAEPEDIMGAC1NLKSIPAVL
          	 :. .   .: :..  .:. ::: :   .. .:.: ..  :....  .:. . .  *.*:

MG01605.1 	ENSTDDRVS-----LSQDG----YECSDRLTLLRSNP-----~-----------------
NCU03023.1	EKPAEEQQGGDLHMTNAAD----DSCVDRLTLLRSMP-----~-----------------
FG00489.1 	QSDDMDNMEIPFPALRERQEGSPPPFQDPLALLRSLP-----~-----------------
AN9160.1  	SHYAMTPWANSSYGCNADN---YLRFIDGYAAYQALPRIARA1GLLAGESETRLPNTMAR
          	.                          *  :  :: *  : : .  :..:.:  ..: : 

MG01605.1 	---------------EAPTVCQVCVVGAGPAGLMMA~ANLARMGVKVQVIDDRADQTPVG
NCU03023.1	---------------EPPVECQVCVVGAGPAGLMLA~CNLGRFGVKVEVVDDRADQTPVG
FG00489.1 	---------------EPPLECQVCVVGAGPAGLMLA~TNLTRYGINVEVIDDRADQTPVG
AN9160.1  	SADHDQGFANIDTDAAPAGETTVVIVGAGPSGLMLA2VNLVRLGTPIVLLDDRPDKTSTG
          	::. ... :. .:.: ..    * :*****:***:*  ** * *  : ::***.*:*..*

MG01605.1 	R2ADGLQPKTLETFRQMRLADPLLQRGVKVYDIAFW~RGTADEPLHRLGREVHYP-PVID
NCU03023.1	R2ADGLQPKTIETFRQMRLADPLLQRGVRVYDIAFW~RSTTDEPLHRLGREIHYP-PIID
FG00489.1 	R2ADGLQPKTIETFRQMRLSDTLLKQGVRVFDIAFW~RSDADQPLRRLGREVHYP-PIID
AN9160.1  	K~ADGIQPKTIETLKQLRLADKLLRDGARIYDISFW0DSTESHPLRRKGRQTHYPDHLVG
          	: ***:****:**::*:**:* **: *.:::**:**  .  ..**:* **: ***. ::.

MG01605.1 	VLDPYILLVHQGMVESLFIEDMKKRGVEVRRNTAFESYSACDNSFGPLQINCRANVTQDK
NCU03023.1	VLDPYILLVHQGMVESLFIEDLKKRGVEVRRNTAFDSYSVCDGNNGPLQVNCLANVTQDR
FG00489.1 	VLDPYILLVHQGMVEGLFLEDMKKRGKEVRRNMAFESYSVPDNKTGPLQINCRANVNQDK
AN9160.1  	ASDPYILLVHQGMLEDVLIDDLAERGVTVTRNSSFLSCSR--NPSKKLDVVYEDQSTGTK
          	. ***********:*.::::*: :**  * ** :* * *   .    *::    : .  :

MG01605.1 	KTILASYLVGC1DGAHSKVRKSIPEARPVGLSQPAVWGVLDG~ELETDFPDIWSKTLVYS
NCU03023.1	KTILTQYLVGC1DGAHSKVRKSIPDAKPIGLSQPSLWGVLDG~ELDTDFPDIWSKTLVYS
FG00489.1 	RSVLTQYLIGC1DGAHSKVRKSIPDVKAVGMSQAAIWGVLDG~ELITNFPDIWSKTLVYS
AN9160.1  	KVIQTEYLVGC~DGARSSVREFIPDAQLEGEMTNASWGVLDG1VIETDFPDLWSKVAVRT
          	: : :.**:** ***:*.**: **:.:  *    : ******  : *:***:***. * :

MG01605.1 	QEHGSILIIPRERNMTRFYIELKSSP--KSDRRELGQEFVMQRAREIMAPFEVGWKYIE~
NCU03023.1	QEHGSILIIPRERNMTRFYIELKSST--KGDRNMLGQEFVMQRAREIMAPFSVKWKYIE~
FG00489.1 	QEHGSILIIPRERNMTRFYIELKAGA--KIDRRDLGQEFMMKRAKAIMAPFRLDWKYVE~
AN9160.1  	HTVGSLLWIPRERGMTRLYVELSATAGERIDKAKATPQYVMERAKEAMKPFSLEWKSIE1
          	:  **:* *****.***:*:**.: ...: *:     :::*:**:  * ** : ** :* 

MG01605.1 	WFGRYQIGQRVASRFADSHNRAFLAGDAS-------HTHSPKAAQGMNTSMHDSWNLAWK
NCU03023.1	WFGRYQIGQRVANRFQDPHTRAFLAGDAS-------HTHSPKAAQGMNTSMHDAWNIGWK
FG00489.1 	WFGRYQVGQRVASRFTDGHLRAFLAGDAS-------HTHSPKSAQGMNTSMHDSWNLSWK
AN9160.1  	WFGNYVVGQRVARHFSDPDYQIFIAGDARPLPSDLFLSLSNRFFRPVTATLRSPPKVQTP
          	***.* :***** :* * . : *:**** . .:.   : * :  : :.::::.. ::   

MG01605.1 	~LNLAVRGLAKPCLLETYENERRKIALDLVNFDYEHANRIAGGDAVALAENFNTNVRFIS
NCU03023.1	~LNFAARGLAKPALLESYEQERRKIALDLVNFDYEHANQIAGGDAVALAENFKTNVRFIS
FG00489.1 	~LNLAVRGLSKPNLLESYEEERRKIALDLVNFDYEHANQIAGGDAIALAENFRTNVRFIS
AN9160.1  	0LNLVARGLASPSLLETYETERRKIANDLIAFDAEHCAAFEAG-EAALARNFDENIRFIS
          	 **:..***:.* ***:** ****** **: ** **.  : .*   ***.**  *:****

MG01605.1 	GVGAEYSENPLN--MHEDQAWVGGEAKPGCLLPPAKVTRYIDLNPVDVQLDIPMLGQFRI
NCU03023.1	GIGAEYGENAIN--MVEPQSWVMGDAKPGCLLPPAKVTRYLDSNPVDVQLDIPMLGQFRI
FG00489.1 	GIGAEYGENAINRPGVGNNQFVMGDAKPGCLLPPAKVTRYIDSNPVDIQLDIPMLGQFRI
AN9160.1  	GVGAEYDASILTQTKVSDAGKGSRRLKPGALLIPAKATRYIDANPVDIQLDVPLLGQFRL
          	*:****. . :. .            ***.** ***.***:* ****:***:*:*****:

MG01605.1 	YLLMWDVHQSRP--FLDMFCNAIASRDSLVSQLSAAASASFALQPRLPSPEDIYWRPERY
NCU03023.1	YLLMWDVHQSRI--FLDGFCAALNSPDSLMNQLSAAANISYAKQPRLPAPEDIYLRPERY
FG00489.1 	YLLMWDVQQSAP--FLQTFCHAIAGKDSFISRLSAAASASYAAQPRASAPEDVYSRPERY
AN9160.1  	YFLIPNVSAAKEKGFLEVVCQILSSPTSILAISAEKAKESYTSRSRGWSATDAYQVPERY
          	*:*: :*  :  ..**: .*  : .  *::   :  *. *:: :.*  :. * *  ****

MG01605.1 	TAVSHLFTFSIIT1TMPRSDIEITDLPPLLQDSRWTFYLDNIPDQDTRGSLCTNKWLGSL
NCU03023.1	TAVSHLFTFGLIT1TMPKSEIEITDLPALFQDSRWTFYLDDIPELDTRGQLCTNKWLGSL
FG00489.1 	TVVSHLFTFGLIT1TMPKTEIEISDLPPLLQDSRWTFYLDDIPDQDTRGSLCTNKWLGSL
AN9160.1  	TTVSEIITLSLIS~GSKREVFEIADLPLALQKSRWTVYLDDVEG-------CIEKWVGEL
          	*.**.::*:.:*:    :  :**:***  :*.****.***::         * :**:*.*

MG01605.1 	GPGEVAIVNVRPDG~YVGSVDRWDSSMDDSGEEAAKWLDMYYDRFLQVPQVDKMKG
NCU03023.1	GPGEVAIVNVRPDG~YVGSIGRWDSSVDDAGEEAAKWLDTYYDRFLQVPPSPAMD-
FG00489.1 	GPGEVAIVNVRPDG~YVGSVGRWDSSVDESGVEAARWLDEYYDGFMQIPTQ-----
AN9160.1  	PETQAGIVLVRPDG2----------------------------NYPRTPNA-----
          	   :..** *****                              : : *
```
